# Psychometric Properties of the Persian Version of the Short Beck Depression Inventory with Iranian Psychiatric Outpatients

**DOI:** 10.1155/2016/8196463

**Published:** 2016-05-17

**Authors:** Mahboubeh Dadfar, Zornitsa Kalibatseva

**Affiliations:** ^1^School of Behavioral Sciences & Mental Health-Tehran Institute of Psychiatry, Iran University of Medical Sciences, International Campus, Tehran, Iran; ^2^Stockton University, Galloway, NJ 08205, USA

## Abstract

The short form of the Beck Depression Inventory (BDI-13) is useful for the screening and assessment of depression in clinical and research settings. The aim of the present study was to investigate the psychometric properties of the Persian (Farsi) version of BDI-13 in an Iranian clinical sample. The sample consisted of 52 Iranian psychiatric outpatients who received services at psychiatric and psychological clinics at the School of Behavioral Sciences & Mental Health-Tehran Institute of Psychiatry, Iran University of Medical Sciences (IUMS) in Tehran, Iran. The study examined the reliability, construct validity, and factor structure of the instrument. The instrument indicated good reliability with Cronbach's alpha of .85 and strong construct validity based on moderate to strong positive correlations with other measures of mental health issues. Using a Principal Component Analysis and Varimax Rotation with Kaiser Normalization, three factors were identified and labeled Affective (F1), Somatic/Vegetative (F2), and Cognitive/Loss of Functioning (F3). The current factor structure suggests that depression is a multidimensional construct in an Iranian clinical sample. This study provides further evidence that the Persian version of the BDI-13 is a psychometrically sound instrument that can be used for clinical and research purposes in Iran.

## 1. Introduction

The assessment of depression with psychometrically valid and reliable instruments ensures the consistent detection of depression symptoms and provides valuable information in facilitating diagnosis and evaluating the treatment process. Instruments that were created and validated in one country are frequently translated and used in other countries. The cross-cultural validation of these translated instruments is important as it allows for comparisons of item endorsements, factor structure, and construct validity of the translated instrument with the original instrument. In addition, it provides valuable information about the cross-cultural presentation of depression and the properties of the instrument with different samples and within different contexts.

One of the most widely used adult self-report depression measures is the Beck Depression Inventory (BDI). It has been utilized in both clinical and research contexts and may often serve as a screening tool in primary care and specialized clinics. The BDI measures depression symptoms and their severity in adolescents and adults (age 13 and older). The original BDI was developed in 1961 [1] and consisted of 21 symptoms and attitudes rated on intensity with different statements ranging from 0 to 3. The first revision, the BDI-IA, was published in 1979 [2] but still contained some issues as it was not assessing all of the symptoms of depression listed in the Diagnostic and Statistical Manual of Mental Disorders. The most recent revision resulted in the current BDI-II version [3], which also consists of 21 items rated from 0 to 3. There have been attempts to create shorter versions of the BDI for screening purposes resulting in the BDI Fast Screen for Medical Patients (BDI-FS [4]) and the BDI-13 [5].

A. T. Beck and R. W. Beck [5] developed the short form of the BDI, the BDI-13, based on self-report and clinician data from 598 patients. The thirteen items in the short form were selected because they demonstrated the highest correlations with the total BDI score and clinician ratings. They assess the following symptoms and attitudes: depressed mood, pessimism, sense of failure, lack of satisfaction, guilt, self-hate, self-harm, social withdrawal, indecisiveness, distorted body image, work difficulty, fatigue, and loss of appetite [6]. Cronbach's alphas of the BDI-13 ranged from .78 to .97 indicating high internal consistency. Correlations with the 21-item version were .94 or higher [7, 8]. These results suggest that the short form is an acceptable substitute for the long form.

The BDI-II has been translated and validated in a number of other languages (e.g., Arabic, Chinese, Dutch, French, German, Japanese, and Italian) and used in research [9]. Despite its excellent psychometric properties, the short version has received less attention cross-culturally and only a few translated and validated versions are available in Chinese [10] and Persian [11]. The Persian version was translated and validated with Iranian college students in 1985 and showed good reliability and validity [11]. Subsequently, Dadsetan and Mansour [12] established norms for the same version in another sample of college students. However, the measure's factor structure and construct validity have not been evaluated with an Iranian clinical sample.

Major depressive disorder (MDD) is a significant public health problem in Iran with prevalence rates ranging from 4.1% in the general population [13] to 12% among women [14]. Depression is one of the most common mental health disorders in Iran and its ubiquity calls for efficient assessment instruments that can be administrated quickly and that demonstrate excellent psychometric properties. The overall goal of the present study was to evaluate the reliability, validity, and factorial structure of the BDI-13 with Iranian psychiatric outpatients. The first aim was to examine the reliability of the BDI-13 and it was hypothesized that the instrument will demonstrate good internal consistency based on previous findings (e.g., [11]). The second aim was to investigate the construct and convergent validity of the BDI-13 and a positive relationship was hypothesized between the BDI-13 and measures of suicidality, hopelessness, death obsession, psychological distress, and mental health problems. The third aim was to explore the factor structure of the BDI-13 and an exploratory factor analysis was used since the previous research on the factor structure of this measure with Iranians is limited.

## 2. Methods

### 2.1. Participants

The sample consisted of 52 Iranian volunteer psychiatric outpatients from psychiatric and psychological clinics at one site in Tehran, Iran: the School of Behavioral Sciences & Mental Health-Tehran Institute of Psychiatry affiliated with Iran University of Medical Sciences. Participants were invited to voluntarily participate in the study after they completed a psychiatric interview with one psychiatrist. Of the initially approached 80 participants, 52 completed the questionnaires. The mean age of patients was 34.89 years (SD = 14.53), 73.1% were female, 59.6% were married, 26.9% were single, and 9.6% were divorced. Half of the sample identified their occupation as housewives, 34.6% were employed, and 55.8% had children. Half of the participants (50%) had a high school diploma, 15.4% had a bachelor's degree, and 15.4% had a graduate degree. More than two-thirds (69.2%) had an average income. The mean duration of diagnosed mental disorder(s) was 10.52 years (SD = 10.99). All of the participants had a psychiatric diagnosis; 26.9% had a depressive disorder and 13.5% had an anxiety disorder. Over a quarter of the sample (28.8%) had a comorbid physical disorder (e.g., diabetes and kidney disease). Approximately half (53.8%) received treatment (93.3% drug therapy, 3.8% drug and psychological therapy). Participants filled out self-report questionnaires during individual sessions. The study was approved by the institution's research and ethics board.

### 2.2. Measures

The* Beck Depression Inventory Short Version* (BDI-S, BDI-13 [5]) consists of 13 items assessing the severity of depression symptoms using statements scored from 0 to 3. The Farsi (Persian) version was used for this study. It was translated and validated with three nonclinical college student samples [11, 12, 15]. The following norms were proposed: normal (0–3); mild depression (4–7); mild to average depression (8–11); average depression (12–15); and severe depression (16–39 [12]). Cronbach alpha from previous studies with Iranian samples ranged from .89 to .94 [15].

The* Beck Suicide Ideation Scale* (BSIS [16]) measures suicidal thinking and consists of 19 items rated on a Likert format ranging from 0 to 2. It shows good reliability and validity in American, Turkish, and Iranian samples [17–19].

The* Beck Hopelessness Scale* (BHS [20]) measures key aspects of hopelessness and is a 20-item self-report inventory rated on a 5-point Likert scale. Khodabakhshi Koolaee et al. [21] translated this measure in Farsi and validated it with an Iranian adolescent sample, reporting good psychometric properties.

The* Death Obsession Scale* (DOS) measures death rumination, death dominance, and death idea repetition. It contains 15 items rated on a 5-point rating scale and demonstrates good validity and reliability in Iranian samples [22–24].

*Kessler Psychological Distress Scale* (K10 [25]) examines psychological distress in 10 self-report items scored on a 5-point Likert scale. In an Iranian sample, its Cronbach alpha was .88 and one-week test-retest reliability was .83 [26].

*General Health Questionnaire-12* (GHQ-12) is a screening tool of mental health scored on a 4-point Likert-type scale. It shows good reliability and validity in Iranian samples as indicated by high positive correlations with similar scales (K10) and Cronbach alpha of .92 [27, 28].

## 3. Results

Table 1 shows means and standard deviations for all BDI-13 items. The most commonly endorsed items were depressed mood/sadness, indecisiveness, sense of failure, fatigability, lack of satisfaction/dissatisfaction, and work inhibition/work difficulty. Next, the instrument's reliability was examined. Cronbach's alpha for this sample was .85, Spearman-Brown coefficient was .70, and Guttman Split-Half coefficient was .67. Interitem correlations ranged from .028 to .690, and the item-total correlations ranged from .454 to .768 (Table 2). These results suggest good internal consistency. Correlations between BDI-13 scores and BSIS, BHS, DOS, K10, and GHQ-12 appear in Table 3. All of the correlations were positive and statistically significant at the .05 level, indicating the measure's good construct validity.

### 3.1. Factor Analysis

The criteria for a factor analysis were evaluated using Kaiser-Meyer-Olkin Measure of Sampling Adequacy (KMO) and Bartlett's Test of Sphericity. The KMO was 0.710, indicating the adequacy of the sample, and Bartlett's Test of Sphericity (292.315, df = 78, *p* < .001) showed that the factor analysis was justified. Using a Principal Component Analysis and Varimax Rotation with Kaiser Normalization three components with eigenvalues greater than one were extracted using SPSS 16 (see Table 4). The scree plot also suggested 3 factors. Factor 1 (items 1–7) was labeled “Affective” and included items such as “I am so sad or unhappy that I can't stand it.” Factor 2 (items 11–13) was labeled “Somatic/Vegetative” and included items such as “I get tired more easily than I used to.” Factor 3 (items 8–10) was labeled “Cognitive/Loss of Functioning” and included items such as “I am worried that I am looking old or unattractive.”

## 4. Discussion

The present study aimed to explore the reliability, validity, and factor structure of the Persian version of the BDI-13 with an Iranian clinical sample. The BDI-13 demonstrated good internal consistency and construct validity in this sample of Iranian psychiatric outpatients. The most commonly endorsed depressive symptoms were items 1 (depressed mood/sadness), 9 (indecisiveness), 3 (sense of failure), 12 (fatigability), 4 (lack of satisfaction/dissatisfaction), and 11 (work inhibition/work difficulty), respectively. Using the norms by Dadsetan and Mansour [12], 17% scored in the normal range, 20.8% were in the mild depression range, 18.9% were in the mild to average depression range, 15.1% were in the average depression range, and 28.3% fell in the severe depression range. These scores indicate that the participants in this study reported higher level of depression symptom severity compared to previous studies [11, 15] that recruited nonclinical college student samples.

All correlations of the BDI-13 with the other measures that are conceptually related to depression were positive and statistically significant. In particular, the measures of suicide ideation, death obsession, general psychological distress, and mental health problems indicated moderate to strong correlations (ranging from .35 to .79) with the BDI-13. These correlations provide strong evidence for the BDI-13's construct validity with an Iranian clinical sample.

The present study identified three components of the BDI-13: Affective, Somatic/Vegetative, and Cognitive/Loss of Functioning. Thus, the construct of depression as measured by the BDI-13 in our sample comprised three dimensions, which is consistent with proposals for the multidimensionality of depression cross-culturally [29]. Our findings differ somewhat from Rajabi's [15] study, which obtained two factors in Iranian university students. In particular, eight items loaded on Factor 1 (1, 2, 3, 4, 5, 6, 7, and 10) labeled “negative emotion towards self” (43.9% of variance) and six items (7, 8, 9, 11, 12, and 13) loaded on Factor 2 labeled “not-enjoying” (8.6% variance; item 7 loaded on both factors). The three factors obtained in this study explained 62.53% of the variance in BDI-13 scores, which is more than the 52.54% explained by Rajabi [15]. One possible explanation for the observed differences between the two studies is the use of a clinical versus a nonclinical sample.

Considering that major depression is one of the most common mental disorders in Iran [30], there is a need for reliable and valid screening measures of depression that can be used with clinical and nonclinical samples. In a community sample in Tehran, the prevalence of major depressive disorder (MDD) was 12.6% among Iranian women and 8.47% among Iranian men [14]. In particular, depression may be a specific concern among Iranian women in primary care settings where patients often get screened for depression [31]. A meta-analysis that examined the epidemiology of MDD in Iran reported consistent findings showing that women were at the higher risk for depression (1.95 OR) than men [13]. Iranian women may be at particular risk for depression after giving birth. Veisani et al. [32] reported that the prevalence of postpartum depression (PPD) in Iran was 25.3% and the history of depression among women with PPD was 45.2%.

The integration of mental health into the primary care health system is an effective approach to screen for depression and has resulted in a reduced use of hospital professional services in Iran. Undoubtedly, the use of brief and psychometrically sound depression instruments will facilitate the detection and treatment of depression. Along with this initiative, the health ministry of Iran is working to reduce stigma associated with mental disorders. There is a comprehensive program, which promotes mental health and aims to educate the public in order to decrease and ideally remove the negative attitudes towards psychiatric patients [33].

The current study provides evidence that the BDI-13 is a reliable and valid instrument for screening depression among samples with psychiatric outpatients in Iran. However, a few limitations need to be noted. First, the study sample was relatively small (*n* = 52), which limits the interpretations of the results and introduces the possibility of overestimating the magnitude of associations. In addition, the study findings may not generalize to other populations, such as psychiatric inpatients or outpatients from other medical clinics. Future research studies need to address these limitations by recruiting larger samples from diverse medical settings, such as primary care, specialty clinics, and inpatient units. Moreover, it will be beneficial to extend this research to other commonly used depression instruments and assess their psychometric properties in other countries in order to establish their cross-cultural validity.

## Figures and Tables

**Table 1:** Means and standard deviations of BDI-13 items.

BDI-13 items	Mean (SD)
(1) Depressed mood/sadness	1.24 (1.03)
(2) Pessimism	.54 (.79)
(3) Sense of failure	1.15 (.98)
(4) Lack of satisfaction/dissatisfaction	.96 (.97)
(5) Guilty feelings	.83 (1.03)
(6) Self-hate/self-dislike	.64 (.85)
(7) Self-punitive wishes/self-harm	.39 (.90)
(8) Social withdrawal	.83 (.91)
(9) Indecisiveness	1.24 (.85)
(10) Distorted body image/self-image	.69 (1.03)
(11) Work inhibition/work difficulty	.92 (.99)
(12) Fatigability	.98 (.84)
(13) Loss of appetite	.43 (.69)
Total score	11.03 (7.55)

**Table 2:** BDI-13 interitem correlations.

Item	1	2	3	4	5	6	7	8	9	10	11	12	13
1	1												
2	.462^*∗ ***∗**^	1											
3	.508^*∗ ***∗**^	.381^*∗∗*^	1										
4	.521^*∗ ***∗**^	.519^*∗ ***∗**^	.542^*∗ ***∗**^	1									
5	.453^*∗ ***∗**^	.581^*∗ ***∗**^	.421^*∗ ***∗**^	.317^*∗*^	1								
6	.556^*∗ ***∗**^	.573^*∗ ***∗**^	.497^*∗*^	.465^*∗ ***∗**^	.690^*∗ ***∗**^	1							
7	.222	.492^*∗ ***∗**^	.426^*∗ ***∗**^	.277^*∗*^	.382^*∗∗*^	.434^*∗ ***∗**^	1						
8	.248	.420^*∗ ***∗**^	.476^*∗ ***∗**^	.250	.539^*∗ ***∗**^	.363^*∗∗*^	.547^**∗∗**^	1					
9	.214	.082	.320^*∗*^	.195	.245	.254	.146	.301^*∗*^	1				
10	.197	.368^*∗∗*^	.386^*∗∗*^	.331^*∗*^	.204	.398^*∗∗*^	.316^*∗*^	.516^*∗ ***∗**^	.524^*∗ ***∗**^	1			
11	.391^*∗∗*^	.077	.265	.292^*∗*^	.230	.328^*∗*^	−.179	.028	.452^*∗ ***∗**^	.258	1		
12	.181	.187	.327^**∗**^	.302^*∗*^	.283^*∗*^	.283^*∗*^	−.091	.170	.327^*∗*^	.193	.547^*∗ ***∗**^	1	
13	.250	.258	.379^*∗∗*^	.138	.266	.267	−.034	.240	.207	.295^**∗**^	.354^*∗ ***∗**^	.179	1
*Total*	*.664* ^*∗∗*^	*.672* ^*∗∗*^	*.748* ^*∗∗*^	*.655* ^**∗∗**^	*.706* ^*∗∗*^	*.768* ^**∗∗**^	*.494* ^*∗∗*^	*.640* ^*∗∗*^	*.535* ^*∗∗*^	*.627* ^*∗∗*^	*.510* ^*∗∗*^	*.483* ^*∗∗*^	*.454* ^*∗∗*^

^*∗∗*^ *p* < .01; ^*∗*^ *p* < .05.

**Table 3:** Descriptive statistics of all scales and correlations with the BDI-13.

Scales	Mean	SD	*α*	*r* with BDI-13
Beck Suicide Ideation Scale (BSIS)	2.78	5.54	0.93	0.49^*∗∗*^
Beck Hopelessness Scale (BHS)	10. 57	2.00	0.64	0.35^*∗*^
Death Obsession Scale (DOS)	27.78	13.68	0.94	0.62^*∗∗*^
Kessler Psychological Distress Scale (K10)	17.00	9.89	0.94	0.79^*∗∗*^
General Health Questionnaire (GHQ-12)	18.00	7.94	0.76	0.75^*∗∗*^

^*∗∗*^ *p* < .01; ^*∗*^ *p* < .05.

**Table 4:** Factor loadings of BDI-13 in Iranian psychiatric outpatients (*N* = 52).

Beck Depression Short Inventory (BDI-13) items	1	2	3
(1) Depressed mood/sadness	.**727**	.317	−.044
(2) Pessimism	.**816**	−.106	.191
(3) Sense of failure	.**580**	.282	.380
(4) Lack of satisfaction/dissatisfaction	.**660**	.284	.099
(5) Guilty feelings	.**721**	.141	.239
(6) Self-hate/self-dislike	.**760**	.229	.232
(7) Self-punitive wishes/self-harm	.**526**	−.392	.**561**
(8) Social withdrawal	.394	−.054	.**737**
(9) Indecisiveness	−.049	.**547**	.**637**
(10) Distorted body image/self-image	.145	.212	.**805**
(11) Work inhibition/work difficulty	.141	.**878**	.010
(12) Fatigability	.184	.**692**	.055
(13) Loss of appetite	.227	.407	.168
Eigenvalue	4.97	1.85	1.30
% of variance	38.26	14.24	10.02
% of total variance	62.53		

Component transformation matrix			
(1) Affective (items 1, 2, 3, 4, 5, 6, and 7)	.78	.36	.50
(2) Somatic (items 11, 12, and 13)	−.30	.93	−.20
(3) Cognitive/Loss of Functioning (items 8, 9, and 10)	−.54	.00	.84
